# Supplementary material for: Impact of Provincial Income Inequality on Parenting Styles in China during COVID-19
Source: Behav Sci (Basel). 2024 Jul 10;14(7):587. doi: 10.3390/bs14070587 (PMC11273907; doi:10.3390/bs14070587)
Supplement: Supplementary file 1 [file behavsci-14-00587-s001.zip › behavsci-3061835-supplementary.pdf]

Supplementary Materials

Impact of Provincial Income Inequality on Parenting Styles in China During COVID-19

Table S1. Gini coefficient and Theil index across Chinese provinces.

Province/region	Gini coefficient	Theil index
Anhui	0.42	0.30
Beijing	0.41	0.27
Chongqing	0.46	0.37
Fujian	0.43	0.31
Gansu	0.42	0.31
Guangdong	0.44	0.33
Guangxi	0.43	0.33
Guizhou	0.48	0.40
Hainan	0.39	0.27
Hebei	0.46	0.36
Heilongjiang	0.38	0.25
Henan	0.41	0.29
Hubei	0.44	0.32
Hunan	0.43	0.31
Inner Mongolia	0.29	0.14
Jiangsu	0.43	0.31
Jiangxi	0.47	0.39
Jilin	0.45	0.35
Liaoning	0.43	0.32
Ningxia	0.48	0.40
Qinghai	0.26	0.12
Shaanxi	0.44	0.33
Shandong	0.47	0.37
Shanghai	0.39	0.24
Shanxi	0.45	0.35
Sichuan	0.46	0.37
Tianjin	0.37	0.23
Xinjiang	0.33	0.18
Xizang	0.42	0.30
Yunnan	0.45	0.35
Zhejiang	0.39	0.25

Table S2. Descriptive statistics for variables included in analyses.

	Mean	SD	Minimum	Maximum	% Missing
**Parenting**					
Demandingness					
Child's homework	3.24	1.316	1	5	3.39
Prevent from watching TV	3.46	1.028	1	5	2.24
Restrict TV shows	3.91	1.001	1	5	5.52
Responsiveness					
Give up watching TV for child's studies	3.57	1.215	1	5	2.16
Discuss school matters with child	3.42	1.052	1	5	6.87
Require child to complete homework	3.19	1.201	1	5	3.35
Autonomy granting					
Understand the reasons	2.61	1.294	1	5	5.33
Explain the rationale	2.91	1.225	1	5	3.32
Encourage compliance and independence	2.79	1.317	1	5	5.24
**Control variables**					
Father's years of education	10.38	3.75	0	22	7.15
Mother's years of education	9.82	4.24	0	21	5.43
Parent CCP	0.23		0	1	0.31
Parental social class					
Waged job	0.65		0	1	0.45
self-employment	0.18		0	1	0.45
agricultural work	0.17		0	1	0.45
Male	0.52		0	1	0
Age	12.35	0.72	12	18	2.28
Rural	0.53		0	1	0
Family size	3.63	1.94	1	13	0
Minority	0.09		0	1	1.01

Note: n = 3,768.

We measured the demandingness using three questions to parents, e.g., "Do you check your child's homework?"; "Do you often prevent or stop your child from watching TV?"; and "Do you regularly restrict the types of TV shows that your child watches?" We measured the responsiveness using three questions to parents: "Do you give up watching TV for your child's studies?"; "Do you often discuss school matters with your children?"; and "Do you require your child to complete their homework?" Finally, we measured the autonomy-granting using four questions to children: "When you make a mistake, your parents seek to understand the reasons, discuss the correct actions with you, and encourage you to learn from the experience"; "Your parents explain the rationale behind their requests, promoting an understanding that encourages compliance and independent judgment"; Your parents support your efforts in tackling tasks, emphasizing the importance of hard work and the value of perseverance"; and "Your parents foster your autonomy by encouraging you to analyze problems and think critically, guiding you toward independent problem-solving."

Table S3. Fit statistics for LCAs modelling the parenting styles excluding respondents who were not parents (N = 3,534).

Class	Loglik	*df*	BIC	Entropy	*p* (LMR-LRT)
2	-6361.8	6659	121904.4	0.85	0.002
3	-60312.5	6598	114364.2	0.91	0.044
4	-57295.2	6523	113761.3	0.81	0.248
5	-55356.3	6454	109982.3	0.79	0.329

Table S4. Impact of Theil index on choosing parenting styles.

	Autonomy granting vs. authoritarian	Autonomy granting vs. average-level undifferentiated	Authoritarian vs. average-level undifferentiated
All participants	0.912	1.684***	2.231***
	(0.323)	(0.191)	(0.221)
Male	0.791*	1.845***	2.579***
	(0.123)	(0.225)	(0.201)
Female	1.108	1.271	1.438**
	(0.225)	(0.291)	(0.128)
Maternal education	0.972	2.282***	2.432***
< 12 years	(0.096)	(0.136)	(0.096)
Maternal education	1.035	1.421**	1.553***
> 12 years	(0.229)	(0.252)	(0.193)
Rural	1.098	**1.452***	1.921***
	(0.285)	**(0.221)**	(0.165)
Urban	1.159	1.115	1.425**
	(0.194)	(0.275)	(0.201)

Note: Exponentiated coefficients; Standard errors in parentheses. Compounding variables have been controlled. * $p < 0.05$, ** $p < 0.01$, *** $p < 0.001$.

One bold value significantly alters the point estimates related to income inequality.

Impact of individual characteristics on the choice of parenting styles.

Table 2 shows paternal education exhibits a modest influence on parenting styles. Each one-year increase in paternal education corresponds to a slight increase in the likelihood of choosing autonomy granting over authoritarian parenting (OR = 1.124*) and autonomy granting over average-level undifferentiated parenting (OR = 1.118*). Additionally, it slightly impacts the preference for authoritarian over average-level undifferentiated parenting (OR = 1.139*). Maternal education demonstrates a stronger influence on parenting styles. Each one-year increase in maternal education significantly increases the likelihood of preferring autonomy granting over authoritarian parenting (OR = 1.218**) and autonomy granting over average-level undifferentiated parenting (OR = 1.114*). Moreover, it impacts the preference for authoritarian over average-level undifferentiated parenting (OR = 1.130*).

Parental CCP membership significantly influences parenting styles. CCP membership is linked to a substantial increase in the likelihood of choosing autonomy granting over authoritarian parenting (OR = 1.579**) and over average-level undifferentiated parenting (OR = 1.427*). However, it does not show the preference for authoritarian over average-level undifferentiated parenting (OR = 1.189).

Regarding parental occupation, wage job holders exhibit a significant increase in the likelihood of choosing autonomy granting over authoritarian parenting (OR = 1.356**) and a substantial increase in the preference for autonomy granting over average-level undifferentiated parenting (OR = 1.709***). However, they show no significant difference in the preference for authoritarian over average-level undifferentiated parenting. Conversely,

self-employed individuals demonstrate a similar pattern, with a significant increase in the likelihood of choosing autonomy granting over authoritarian parenting (OR = 1.304**) and a substantial increase in the preference for autonomy granting over average-level undifferentiated parenting (OR = 1.575***). Again, no significant difference is observed in the preference for authoritarian over average-level undifferentiated parenting.

Gender demonstrates varying influences on parenting styles. Male gender does not significantly impact the preference for autonomy granting over authoritarian parenting (OR = 0.982), yet it significantly increases the likelihood of choosing autonomy granting over average-level undifferentiated parenting (OR = 1.309***) and preferring authoritarian over average-level undifferentiated parenting (OR = 1.445***).

Child age corresponds to a significant increase in the preference for autonomy granting over average-level undifferentiated parenting (OR = 1.280**) and the preference for authoritarian over average-level undifferentiated parenting (OR = 1.417***). However, child age does not exhibit a statistically significant association with the preference for autonomy granting over authoritarian parenting (OR = 1.102).

Rural residence significantly impacts parenting styles, indicating potential contextual influences. Residing in rural areas is associated with a decreased likelihood of choosing autonomy granting over authoritarian parenting (OR = 0.647***) and no significant difference in the preference for autonomy granting over average-level undifferentiated parenting (OR = 1.123). However, it significantly increases the likelihood of preferring authoritarian over average-level undifferentiated parenting (OR = 1.476***).

Family size demonstrates a significant influence on parenting styles. One more family member is associated with a substantial decrease in the likelihood of selecting autonomy granting over authoritarian parenting (OR = 0.519***) and a significant decrease in the preference for autonomy granting over average-level undifferentiated parenting (OR = 0.677***). However, it does not significantly impact the preference for authoritarian over average-level undifferentiated parenting (OR = 0.965).

Minority status significantly influences parenting styles. Belonging to a minority group is associated with a decreased likelihood of choosing autonomy granting over authoritarian parenting (OR = 0.433***) and a decreased likelihood over average-level undifferentiated parenting (OR = 0.539**). However, it does not significantly impact the preference for authoritarian over average-level undifferentiated parenting (OR = 1.186)

Based on the results, we see the important roles of parental factors. It is evident that parental education, particularly maternal education, has a significant influence on the adoption of autonomy granting parenting practices. Educational systems often propagate certain values, such as discipline, reasoning, and independence, which are congruent with autonomy granting parenting characteristics (Steinberg et al., 1992) [91]. Educated parents, drawing upon their academic experiences, may be more attuned to the long-term benefits of an authoritarian style.

The tendency towards authoritarian and autonomy granting parenting in families where at least one parent is a member of the CCP can be linked to multiple interconnected factors within China. The CCP emphasizes the development of its members and the strict observance of party discipline, advocating for values like self-regulation while rejecting indulgence and lavishness (Du & Li, 2023) [9]. Furthermore, the process of selecting CCP members is notably rigorous and selective, attracting many individuals from elite backgrounds who possess socio-economic benefits not available to non-members (Zeng, 2016) [92]. Membership in the party thus involves not just access to resources but also a commitment to certain ideals, both of which are influential in setting the standards for parenting behaviors and attitudes.

The parental occupational class is a substantial role in predicting parenting practices. Similar to prior studies (e.g., [9]), parents with wage jobs and self-employed parents, as opposed to agricultural workers, are more inclined towards autonomy granting parenting. This preference may stem from several factors associated with different occupational classes. For instance, individuals in wage jobs often possess higher levels of education [93], which can lead to greater awareness of autonomy granting parenting principles and their benefits for child development [94]. Furthermore, the nature of self-employment may require individuals to exercise leadership, decision-making, and communication skills [95], which could translate into a preference for autonomy granting parenting characterized by clear expectations and open communication.

For other individual characteristics, we also obtain important findings. For example, a child's gender influences parenting styles, with parents more likely to adopt autonomy granting and authoritarian styles and less likely to choose average-level undifferentiated parenting when their child is male instead of female. This may stem from the traditional Chinese preference for sons, which places greater importance on boys, prompting parents to exhibit increased demands, responsibility, and nurturing behavior towards their sons [78]. As children age, parents tend to favor autonomy granting and authoritarian styles over average-level undifferentiated parenting. This shift reflects a dual dynamic: some parents emphasize autonomy and independence, while others perceive their older children as better equipped for increased responsibilities, leading to a transition towards authoritarian parenting. In terms of *hukou* status, rural parents are more likely to adopt average-level undifferentiated parenting compared to urbanites. This is inconsistent with the prior finding that a rural-urban divide in parenting styles [81], indicating that rural parents in China are more inclined to uphold traditional values and beliefs, such as valuing strictness and control, which are characteristics of authoritarian parenting. One potential reason is that cultural shifts and modernization may lead to evolving parenting norms in rural areas, potentially contributing to the adoption of different parenting styles among rural parents. Furthermore, our results indicate that family size impacts parenting styles, with few siblings significantly predicting authoritarian parenting practices. Regarding minority status, our findings suggest that minority parents are less likely to adopt authoritarian and more likely to employ average-level undifferentiated parenting styles compared to *Han* parents.
